# Supplementary material for: Sparse genetic tracing reveals regionally specific functional organization of mammalian nociceptors
Source: eLife. 2017 Oct 12;6:e29507. doi: 10.7554/eLife.29507 (PMC5648527; doi:10.7554/eLife.29507)
Supplement: Figure 3—source data 1. — Data pooled from nine 3pw animals. Asterisk (*) indicates a significant difference from other regions (p<0.05, one-way ANOVA with Tukey’s multiple comparisons test). [file elife-29507-fig3-data1.docx]

| **Region** | n | **Avg area (mm^2^)** | SE | Min area (mm^2^) | Max area (mm^2^) |
| --- | --- | --- | --- | --- | --- |
| Plantar hindpaw (glabrous) | 22 | **0.30** | 0.10 | 0.08 | 0.86 |
| Dorsal hindpaw | 23 | **0.29** | 0.04 | 0.15 | 0.54 |
| Proximal hindlimb | 23 | **0.17** | 0.03 | 0.14 | 0.32 |
| Dorsal forepaw | 9 | **0.26** | 0.07 | 0.11 | 0.45 |
| Proximal forelimb | 3 | **0.14** | 0.01 | 0.13 | 0.14 |
| Trunk | 76 | **0.28** | 0.05 | 0.10 | 0.71 |
| Top of head | 6 | **0.14** | 0.04 | 0.10 | 0.20 |
| Ear* | 11 | **0.47** | 0.11 | 0.22 | 0.93 |

**Figure 3 – source data 1. Summary of peripheral terminals of sparsely labeled Mrgprd^+^ non-peptidergic nociceptors.** Data pooled from nine 3pw animals. Asterisk (*) indicates a significant difference from other regions (*p*<0.05, one-way ANOVA with Tukey’s multiple comparisons test).
