# Supplementary material for: Sparse genetic tracing reveals regionally specific functional organization of mammalian nociceptors
Source: eLife. 2017 Oct 12;6:e29507. doi: 10.7554/eLife.29507 (PMC5648527; doi:10.7554/eLife.29507)
Supplement: Figure 3—source data 2. — Side (right or left) of injection locations were alternated between animals. Asterisks (**) indicate missing tissue. [file elife-29507-fig3-data2.docx]

| **Animal** | **Proximal Hindlimb** | | **Plantar Hindpaw** | |
| --- | --- | --- | --- | --- |
|  | DiI^+^ Skin Area (mm^2^) | DiI/EGFP double^+^ DRG neurons | DiI^+^ Skin Area (mm^2^) | DiI/EGFP double^+^ DRG neurons |
| 1 | 1.59 | 173 | 5.54 | 128 |
| 2 | 0.89 | 49 | 7.20 | 163 |
| 3 | 2.32 | 75 | 10.54 | 196 |
| 4 | 11.26 | 245 | 4.16 | 149 |
| 5 | 0.58 | 117 | 4.56 | 71 |
| 6 | 0.77 | 182 | 8.46 | 83 |
| 7 | ** | ** | 12.95 | 274 |
| 8 | 2.19 | 60 | ** | ** |

**Figure 3 – source data 2. Retrograde DiI^+^ labeling of nociceptors in *Mrgprd^EGFPf^* mice.** Side (right or left) of injection locations were alternated between animals. Asterisks (**) indicate missing tissue.
