# Supplementary material for: Sparse genetic tracing reveals regionally specific functional organization of mammalian nociceptors
Source: eLife. 2017 Oct 12;6:e29507. doi: 10.7554/eLife.29507 (PMC5648527; doi:10.7554/eLife.29507)
Supplement: Figure 4—source data 1. — Round and long terminals were defined by W/H ratios (round = W/H ratio >0.2, long = W/H ratio <0.2). Data pooled from seven 3pw animals. [file elife-29507-fig4-data1.docx]

|  | **Round** | | | | | **Long** | | | | |
| --- | --- | --- | --- | --- | --- | --- | --- | --- | --- | --- |
|  | n | **Height (µm)** | SE | **Width (µm)** | SE | n | **Height (µm)** | SE | **Width (µm)** | SE |
| Cervical | 31 | **145** | 28 | **49** | 11 | 45 | **259** | 56 | **28** | 5 |
| Thoracic | 3 | **155** | 6 | **37** | 6 | 81 | **334** | 61 | **24** | 4 |
| Lumbar | 99 | **157** | 20 | **48** | 5 | 83 | **274** | 32 | **28** | 3 |
| Sacral | 19 | **162** | 22 | **51** | 7 | 7 | **197** | 19 | **35** | 3 |

**Figure 4 – source data 1. Summary of non-peptidergic nociceptor central arbor height and width measurements.** Round and long terminals were defined by W/H ratios (round = W/H ratio >0.2, long = W/H ratio <0.2). Data pooled from seven 3pw animals.
