# Supplementary material for: Sparse genetic tracing reveals regionally specific functional organization of mammalian nociceptors
Source: eLife. 2017 Oct 12;6:e29507. doi: 10.7554/eLife.29507 (PMC5648527; doi:10.7554/eLife.29507)
Supplement: Figure 7—source data 1. — Patch clamp recordings were taken from either transverse or sagittal DH slices, as indicated. Responses were classified as mono- or polysynaptic (see text). Shaded boxes show the response of the majority (>50%) of recorded cells. [file elife-29507-fig7-data1.docx]

*Mrgprd^CreERT2^*; *Rosa^ChR2-EYFP/ChR2-EYFP^* (ChR2 Homozygous)

|  | *Transverse* | | | |
| --- | --- | --- | --- | --- |
|  | *n* | Mono  EPSC_L_ | Poly  EPSC_L_ | Non-  Responsive |
| Medial  Lumbar | 15 | 11 (73.3%) | 3  (20%) | 1  (6.7%) |
| Lateral  Lumbar | 18 | 16  (88.9%) | 1  (5.6%) | 1  (5.6%) |

*Mrgprd^CreERT2^*; *Rosa^ChR2-EYFP/+^* (ChR2 Heterozygous)

|  | *Transverse* | | | |
| --- | --- | --- | --- | --- |
|  | *n* | Mono EPSC_L_ | Poly  EPSC_L_ | Non-responsive |
| Medial  Lumbar | 17 | 15  (88.2%) | 2  (11.8%) | 0  (0%) |
| Lateral  Lumbar | 19 | 2  (10.3%) | 10  (52.6%) | 7  (36.8%) |
| Thoracic | 11 | 1  (9.1%) | 6  (54.4%) | 4  (36.4%) |
|  | *Sagittal* | | | |
|  | *n* | Mono EPSC_L_ | Poly  EPSC_L_ | Non-responsive |
| Medial  Lumbar | 13 | 9  (69.2%) | 3  (23.1%) | 1  (7.7%) |
| Lateral  Lumbar | 16 | 0  (0%) | 3  (18.8%) | 13  (81.3%) |
| Thoracic | 12 | 1  (8.3%) | 3  (25.0%) | 8  (66.7%) |

**Figure 7 – source data 1. Summary of incidences of light-induced excitatory postsynaptic current (EPSC_L_) responses recorded from layer II neurons in *Mrgprd^CreERT2^*; *Rosa^ChR2-EYFP^* homozygous and heterozygous mice.** Patch clamp recordings were taken from either transverse or sagittal DH slices, as indicated. Responses were classified as mono- or polysynaptic (see text). Shaded boxes show the response of the majority (>50%) of recorded cells.
