# Supplementary material for: Sparse genetic tracing reveals regionally specific functional organization of mammalian nociceptors
Source: eLife. 2017 Oct 12;6:e29507. doi: 10.7554/eLife.29507 (PMC5648527; doi:10.7554/eLife.29507)
Supplement: Transparent reporting form [file elife-29507-transrepform.pdf]

eLIFE
1st Floor
24 Hills Road
Cambridge CB2 1JP, UK
P 01223 855340
W elifesciences.org
T @elife

eLife's transparent reporting form

We encourage authors to provide detailed information *within their submission* to facilitate the interpretation and replication of experiments. If you have any questions, please contact us: editorial@elifesciences.org.

Sample-size estimation

- You should state whether an appropriate sample size was computed when the study was being designed
- You should state the statistical method of sample size computation and any required assumptions
- If no explicit power analysis was used, you should describe how you decided what sample (replicate) size (number) to use

Please outline where this information can be found within the submission (e.g., page numbers or figure legends), or explain why this information doesn't apply to your submission:

We did not perform a power analysis prior to undertaking the study. For most histological experiments, a minimum sample size of 3 animals, and 4 to 6 sections per animal, was chosen to ensure reproducibility, which is consistent with similar experiments published by many labs. For whole mount spinal cord AP color reaction, a minimum sample size of 3 animals and more than 100 AP+ neurons, were quantified. For physiological recording experiments, cells were recorded from a minimum of three animals, yielding at least 10 to 15 cells for each condition. For behavior assays, 6 to 10 animals were used based on previous publications. The animal and cell numbers are shown in each figure legend.

Replicates

- You should report how often each experiment was performed
- You should include a definition of biological versus technical replication
- The data obtained should be provided and sufficient information should be provided to indicate the number of independent biological and/or technical replicates
- If you encountered any outliers, you should describe how these were handled
- Criteria for exclusion/inclusion of data should be clearly stated
- High-throughput sequence data should be uploaded before submission, with a private link for reviewers provided (these are available from both GEO and ArrayExpress)

eLife Sciences Publications, Ltd is a limited liability non-profit non-stock corporation incorporated in the State of Delaware, USA, with company number 5030732, and is registered in the UK with company number FC030576 and branch number BR015634 at the address 1st Floor, 24 Hills Road, Cambridge CB2 1JP | August 2014

Please outline where this information can be found within the submission (e.g., page numbers or figure legends), or explain why this information doesn't apply to your submission:

N numbers are provided in the text when quantifications are mentioned and are stated in figures, figure legends, tables, and table legends. The definitions of a given n value (i.e. arbors vs. animals) is stated along with the value, and the definition of n values for section histology experiments is given in the Methods section on page 30. We did not exclude any data.

Statistical reporting

- Statistical analysis methods should be described and justified
- Raw data should be presented in figures whenever informative to do so (typically when N per group is less than 10)
- For each experiment, you should identify the statistical tests used, exact values of N, definitions of center, methods of multiple test correction, and dispersion and precision measures (e.g., mean, median, SD, SEM, confidence intervals; and, for the major substantive results, a measure of effect size (e.g., Pearson's r, Cohen's d)
- Report exact p-values wherever possible alongside the summary statistics and 95% confidence intervals. These should be reported for all key questions and not only when the p-value is less than 0.05.

Please outline where this information can be found within the submission (e.g., page numbers or figure legends), or explain why this information doesn't apply to your submission:

Statistical tests are stated in the Results section as well as in Figure or Table legends, and are described in the Methods section (pages 30-31).

(For large datasets, or papers with a very large number of statistical tests, you may upload a single table file with tests, Ns, etc., with reference to page numbers in the manuscript.)

Additional data files ("source data")

- We encourage you to upload relevant additional data files, such as numerical data that are represented as a graph in a figure, or as a summary table
- Where provided, these should be in the most useful format, and they can be uploaded as "Source data" files linked to a main figure or table
- Include model definition files including the full list of parameters used
- Include code used for data analysis (e.g., R, MatLab)
- Avoid stating that data files are "available upon request"

Please indicate the figures or tables for which source data files have been provided:

Summary tables for key experiments (Tables 1-4) are provided in the manuscript.
